# Preksha Dhyana meditation modulates the serum metabolome in healthy and meditation-naïve participants

**DOI:** 10.3389/fmolb.2026.1741802

**Published:** 2026-04-15

**Authors:** Bassam Abomoelak, Nidhi Kapoor, Mary Schreck, Samani U. Pragya, Samani C. Pragya, Neelam Mehta, José Carlos Bozelli, Meera Shanmuganathan, Zachary Kroezen, Philip Britz-Mckibbin, Parvin Uddin, Pushya Veeramachaneni, Naina Mehta, Ray Prather, Arpit Mehta, Devendra Mehta

**Affiliations:** 1 Gastrointestinal Translational Laboratory, Arnold Palmer Hospital for Children, Orlando, FL, United States; 2 Department of Religions and Philosophies, University of London, London, United Kingdom; 3 Department of Biostatistics, Robert Stempel College of Public Health and Social Work, Florida International University, Miami, FL, United States; 4 Department of Anesthesiology, Orlando Health, Orlando, FL, United States; 5 Department of Chemistry and Chemical Biology, McMaster University, Hamilton, ON, Canada; 6 College of Law, Florida International University, Miami, FL, United States; 7 Legs Therapy, Orlando, FL, United States; 8 Embry-Riddle Aeronautical University, Dayton Beach, FL, United States; 9 Knight Foundation School of Computing and Information Sciences, Florida International University, Miami, FL, United States

**Keywords:** cellular pathways, correlation, DNA methylation, lipidomics, metabolomics, Preksha Dhyana meditation, serum

## Abstract

The impact of various meditation interventions on modifying human health outcomes has been demonstrated at both cognitive and cellular levels. Preksha Dhyana (PD) meditation has previously shown effects on cognitive skills, transcriptome, and the DNA methylome in healthy, novice college students. In this study, we performed a cross-platform metabolomic and lipidomic analysis of serum samples collected from 38 healthy, meditation-naïve participants and five age-matched participants who served as controls. Serum samples were collected from both groups, at baseline (pre-meditation) and after an 8-week PD intervention. Analysis was conducted using capillary electrophoresis and reversed-phase liquid chromatography coupled to electrospray ionization–mass spectrometry, and higher serum concentration levels for four circulating metabolites, namely, hypoxanthine, oxoproline, choline, and cystine, were identified along with a series of lysophosphatidylcholine (LPC) and lysophosphatidylethanolamine (LPE) species (LPC 20:0, LPC 18:0, LPC 18:1, LPC 16:0, LPE 18:0, LPE 18:1, and LPE 22:6), following the PD intervention that satisfied a Bonferroni correction (p < 0.05, FDR). Pathway enrichment analysis revealed that these metabolites and lipids are primarily associated with the purine degradation cycle, glycerophospholipid metabolism, and glutathione metabolism, which are critical for maintaining cellular energy balance, preserving membrane integrity, and protecting against oxidative stress. A multi-omics analysis with Data Integration Analysis for Biomarker discovery using Latent approaches for Omics studies (DIABLO) revealed positive and negative correlations between these metabolites/lipids, DNA methylated sites (DMSs), and cognitive outcomes (r > 0.5). Overall, our findings suggest that the wellbeing experienced after PD meditation might be associated with bioactive metabolites and lipids, which may also function as epigenetic modifiers regulating gene expression and biological pathways.

**Clinical Trial Registration:**

ClinicalTrials.gov, Identifier NCT03779269.

## Introduction

Meditation helps reduce stress, depression, and anxiety by training the mind to respond positively to stressful situations and, therefore, triggering the body’s relaxation response (Goyal et al., 2014; Hoge et al., 2013). Moreover, meditation contributes to a calm mental state, allowing the body to repair itself, and prevents the deleterious physiological effects of chronic stress, such as hypertension and immune suppression (Spruill, 2010; Segerstrom and Miller, 2004). Studies suggest that gene expression and epigenetic changes play a role in post-meditation relief feelings and that relaxation induces temporal transcriptomic alterations in inflammatory and energy metabolism pathways (Bhasin et al., 2013; Qu et al., 2013). Hence, several meditation techniques have been implemented as clinical intervention tools in combating depression, attention-deficit hyperactive disorder, pain management, and drug addiction (Zylowska et al., 2008; Speca et al., 2000; Kuntz et al., 2018).

Mass spectrometry-based metabolomics provides new insights into the mechanisms of human health and disease based on the comprehensive analysis of metabolites in complex biological samples (Cajka and Fiehn, 2016; Chi et al., 2024). So far, most meditation interventions have relied on a targeted analysis of hormonal, inflammatory, lipid, and protein biomarkers to assess treatment responses, such as changes in cortisol, cytokine, endocannabinoid, triglyceride, and brain-derived neurotrophic factor levels (Gardi et al., 2022; Sadhasivam et al., 2020; Xue et al., 2018). Until now, untargeted metabolomic studies involving specific meditation practices remain limited. Recently, a serum metabolomics study of a mindfulness intervention examined the effects of short-term integrative body–mind training relative to relaxation training as an active control to improve the physical and mental wellbeing of participants (Tang et al., 2025). The study revealed distinct temporal changes in serum metabolites following 10 sessions of integrative body–mind training compared to relaxation training, such as increases in fumarate, glycine, glutamate, and tetrahexosylceramide levels. In addition, a plasma lipidomic study was conducted on residents completing an 8-day Samyama meditation/yoga retreat, which revealed alterations in specific lipid signatures, including acylglycine, phosphatidylcholine, phosphatidylethanolamine, and plasmalogen species, along with changes associated with cellular anandamide, vascular relaxation, anti-inflammatory biomarkers, and analgesia (Vishnubhotla et al., 2022). Metabolic changes in blood glucose and lipid metabolism between healthy and depressive participants following an 8-week mindfulness intervention in China were also confirmed, but the analysis was limited to standard clinical blood markers rather than a full untargeted metabolomic assessment (Xue et al., 2018). However, meditation protocols differ widely in their implementation, duration of practice, and participant selection, which makes direct comparison of metabolomic studies challenging, especially if confounded by dietary changes, and/or physical exercises (Tang et al., 2022; Fan et al., 2024; Hwangbo et al., 2022; Jasbi et al., 2023).

PD meditation blends yoga and meditation through guided physical and mental practices designed to deepen self-awareness. It incorporates preparatory exercises and mantra-chanting to focus attention and promote mental and physical health. Our previous study involving college student participants who experienced different stress factors throughout their school year showed an improvement in cognition, attention, and overall wellbeing following PD meditation (Samani et al., 2021). PD meditation also induced significant effects on gene expression and DNA methylation (Pragya et al., 2021; Abomoelak et al., 2022; Pragya et al., 2023). In addition, PD was found effective in reducing adverse clinical symptoms in children with functional abdominal pain disorders (FAPDs), including abdominal pain, stress, and vomiting (Vijay Mehta et al., 2021).

PD studies have primarily measured changes in the transcriptome and microbiome of participants, and recently, a link between meditation and the immune system, human microbiome, and epigenetics has also been reported (Househam et al., 2017). However, the impact of PD on adaptive metabolic phenotype changes in study participants remains unexplored so far. Furthermore, the relationship between changes in circulating metabolite levels and lipids with DNA methylation and cognitive skills after meditation has not yet been investigated. These correlations are needed to derive a deeper understanding of the effects of PD meditation to validate its potential as an early clinical intervention for the treatment or prevention of stress-related chronic diseases.

To address this knowledge gap, in this study, we conducted a cross-platform metabolomic and lipidomic analysis in an untargeted manner to identify key serum metabolites and lipid species with altered levels after 8 weeks of PD in 38 healthy college students. Treatment-responsive metabolites and lipids in circulation were then correlated with previously measured cognitive scores and 470 differentially expressed DNA methylated sites. This study aims to identify putative epigenetic modifiers that may regulate gene expression and be associated with improved cognitive outcomes following PD intervention.

## Methods

### Participants and study design

The study was approved by the Florida International University (FIU) Ethics Committee and Institutional Review Board (IRB-17-0108-CR02). Participants were healthy FIU college students, including 38 individuals (9 male and 29 female individuals) with a median age of 23.5 years old. Five age-matched students with a median age of 22 years (four male individuals and one female individual) served as controls. Inclusion criteria required participants to be healthy, enrolled FIU students with no prior meditation experience; exclusion criteria included patients with chronic health conditions or blood disorders. To ensure meaningful exposure to the intervention and reduce variability in outcomes, participants were required to attend more than 80% of sessions. All participants provided written consent and were assigned IRB-compliant identification numbers.

The study was conducted over six semesters, with new participants enrolled each term and assigned to intervention or control groups based on class-schedule availability, ensuring consistent access to meditation rooms and the lead instructor. The intervention consisted of a 25-minute guided meditation session three times per week for 8 weeks, with brief relaxation periods before and after each session. Pre- and post-tests were administered at baseline and week 8, and adherence was assessed three times per session by measuring the duration of buzzing during *mahapran*, a sustained humming produced after a deep inhalation with the eyes closed. Meditation protocols, participant assignments, data collection, and cognitive assessments were described previously.

The five control participants provided blood samples at baseline and after 8 weeks but did not participate in the PD intervention. All serum samples were otherwise stored at −80 °C until analysis. Details on blood collection, DNA methylation data, and other protocols are described elsewhere (Pragya et al., 2023).

### Chemicals and reagents

LC–MS-grade water, methanol, acetonitrile, HPLC plus-grade isopropanol, dichloromethane, ammonium acetate, and deuterated internal standards, including lauric acid-d3 (LA-d3,), stearic acid-d35 (SA-d35), and EquiSPLASH Lipodomix™ comprising a mixture of 13 different lipid classes, were purchased from Sigma-Aldrich (St. Louis, MO, USA). Methyl-*tert*-butyl ether (MTBE) and hydrochloric acid were purchased from Caledon laboratories Ltd. (Georgetown, ON, Canada). The 11 polar metabolite internal standards used in serum metabolomic analyses, including ornithine-^15^N_2_, choline-d9, creatinine-d3, carnitine-d3, γ-aminobutyric acid-d6, histidine-15N, 3-methylhistidine-d3, and trimethylamine-N-oxide-d9, were purchased from Cambridge Isotopes Laboratories Inc. (Tewksbury, MA, USA), while 3-fluorophenylalanine (F-Phe), 4-chlorotyrosine (Cl-Tyr), and 3-fluorotyrosine (F-Tyr) were obtained from Sigma-Aldrich Inc. All reagents were used as received without further purification.

### Serum metabolomics by multisegment injection–capillary electrophoresis–mass spectrometry

Untargeted analysis of polar/ionic metabolites was performed using an Agilent 7100 capillary electrophoresis (CE) system coupled to an Agilent 6230 time-of-flight (TOF)–MS system and coaxial sheath liquid interface (Agilent Technologies Inc., Mississauga, ON, Canada). Serum metabolomic analysis was performed through multisegment injection–capillary electrophoresis–mass spectrometry (MSI-CE-MS) under two configurations with full-scan data acquisition for cationic and anionic metabolites in positive and negative ion modes using a standardized data workflow for molecular feature annotation and metabolite authentication (Shanmuganathan et al., 2021a). In brief, all serum samples were first diluted five-fold in deionized water containing a mixture of internal standards prior to their ultrafiltration to remove proteins using pre-washed Nanosep 3-kDa MWCO filters (Millipore Sigma Inc., Oakville, ON, Canada). Unmodified polyimide-coated fused-silica capillaries with a total length of 135 cm, an inner diameter of 50 μm, and an outer diameter of 360 μm were used (Polymicro Technologies Inc, AZ, USA). High-throughput metabolomic analysis was performed via a serial injection format in MSI-CE-MS comprising an alternating hydrodynamic injection of a sample (100 mbar for 5 s), followed by an electrokinetic injection (30 kV for 75 s) of the background electrolyte (BGE). This sequence was repeated 12 more times until a total of 13 samples (e.g., blank, calibrant, and/or serum filtrate) were introduced onto the capillary, after which a voltage of 30 kV was applied for the duration of the electrophoretic separation (Saoi et al., 2019; Shanmuganathan et al., 2021b). All serum metabolites were annotated based on their accurate mass: relative migration time (*m/z*:RMT) under positive (p) or negative (n) ion mode detection, and their ion responses were normalized to an internal standard (20 μmol/L, 4-chlorotyrosine, Cl-Tyr or naphthalene monosulfonic acid, NMS). Most serum metabolites were unambiguously identified (level 1) after spiking authentic standards into serum filtrate samples. The top-ranked metabolites of significance were also quantified (μmol/L) using an external calibration curve by MSI-CE-MS. Otherwise, unknown metabolites were annotated based on their most likely molecular formula. Overall, 66 polar/ionic metabolites, comprising 47 metabolites that were quantified and unambiguously identified (level 1) and 19 annotated molecular ions with unknown chemical structures (level 3), were consistently measured in serum filtrate samples using MSI-CE-MS, which also satisfied frequency (>75% of all serum samples analyzed) and technical precision (mean CV < 30%) filters following repeat analysis of a pooled quality control (QC) sample (n = 11).

### Serum lipidomics by reversed-phase liquid chromatography–mass spectrometry

Comprehensive lipidomics analyses were performed on serum ether extracts using an Agilent 1290 Infinity II ultra-high-performance liquid chromatography (UHPLC) system coupled to an Agilent 6230 TOF–MS system with a dual spray Agilent Jet Stream (DualAJS) electron spray ionization (ESI) ion source. Chromatographic separation was performed on an InfinityLab Poroshell 120 EC-C18 column (4.6 × 100 mm, 2.7 μm) preceded by an Infinity Lab Poroshell 120 EC-C18 guard column (4.6 × 5 mm, 2.7 μm). The mobile phase consisted of (A) 60:40 vol acetonitrile:water with 10 mmol/L ammonium acetate and (B) 90:9:1 (vol) isopropanol: acetonitrile:water with 10 mmol/L ammonium acetate. The gradient elution program was as follows: 0 min, 15% B; 1.33 min, 30% B; 1.67 min, 48% B; 7.33 min, 82% B; 7.67 min, 99% B; 8 min, 99% B; 8.07 min, 15% B; and 10 min, 15% B. The flow rate was maintained at 0.6 mL/min, and the column oven temperature was set to 40 °C. The Dual AJS ion source was operated in the negative ionization mode with full-scan data acquisition using the following parameters: drying gas temperature of 300 °C, a drying gas flow rate of 11 L/min, nebulizer pressure of 30 psi, sheath gas temperature of 350 °C, a sheath gas flow rate of 11 L/min, capillary voltage of 3.5 kV, nozzle voltage of 1.5 kV, and fragmentor voltage of 120 V. Mass spectra were acquired at a speed of 1 spectrum/s over a mass range of 50–1,700 *m/z* using the extended dynamic range setting (2 GHz). Lipids were extracted from 20 µL of serum using a modified Maytash protocol with methyl-*tert*-butyl ether (MTBE), as described previously (Azab et al., 2019; Ly et al., 2023). In brief, a 20-µL serum aliquot was transferred to a glass vial pre-rinsed with dichloromethane. Then, 225 µL of methanol, 20 µL of 667 ng/mL EquiSPLASH Lipidomix solution, 12.5 µL of 1 M HCl, and 750 µL of MTBE containing 4 μmol/L of FA 18:2-d3 and of FA 18:0-d35 were added sequentially. The vial was capped, and the mixture was shaken vigorously for 25 min at room temperature. Phase separation was induced by adding 188 µL of LC-MS-grade water, followed by another shaking step for 2 min, and centrifugation at 3,200 *g* for 15 min at 4 °C. The upper organic phase (600 µL) was carefully collected and transferred to an amber glass vial pre-rinsed with dichloromethane. The collected organic phase was then dried under a gentle stream of nitrogen gas, the vials were capped, and dried lipid extracts were stored at −80 °C. Prior to LC-MS analysis, the dried lipid extracts were reconstituted in 20 µL of methanol, shaken for 15 min, and transferred to a 250-µL glass insert in an amber autosampler vial. Procedure blanks were also prepared by extracting ultrapure water with and without added internal standards.

To ensure data quality and reliability, several quality control measures were implemented: (i) the sample injection sequence was randomized to minimize carry-over effects and systematic biases; (ii) eight pooled QC samples were injected at the beginning of the analytical sequence to equilibrate the LC–MS system; (iii) QC samples were injected periodically throughout the run after every 20 experimental samples to monitor system stability and performance; (iv) procedure blanks were analyzed throughout the run after every 20 experimental samples to assess potential contamination from solvents or consumables; (v) the peak shape and intensity of the spiked internal standards were carefully monitored throughout the analysis to assess extraction efficiency and instrument stability; and (vi) the mass accuracy of the internal standards was continuously monitored during the LC-MS runs to ensure instrument performance. Raw data files were converted to the mzML format using ProteoWizard (Adusumilli and Mallick, 2017), whereas peak picking, alignment, and integration were performed using MZmine 4.5 with the following parameters: intra-sample retention time (RT) tolerance of 0.04 min, sample-to-sample RT tolerance of 0.10 min, intra-sample tolerance of 0.0015 *m/z* or 3 ppm, sample-to-sample tolerance of 0.0040 *m/z* or 8 ppm, noise threshold at 500 ion counts, minimum feature height of 1,000 ion counts, and a Savitzky–Golay smoothing algorithm with a window width of five data points for RT smoothing (Schmid et al., 2023). The resulting feature list was subjected to blank subtraction, and features with a detection frequency lower than 75% in the QC sample were excluded to remove inconsistent or spurious signals. Lipid annotation was achieved by matching accurate mass and retention time to the CEU Mass Mediator database as well as evaluating a linear change in RT profiles among a homologous series of lipids (Gil-de-la-Fuente et al., 2019). Overall, after a blank subtraction, 131 serum lipids (from six major classes) were annotated based on their sum notation and consistently measured in this study when using reversed-phase LC-MS under negative ion mode detection, which also satisfied frequency (>75% of serum samples) and technical precision (mean CV < 30%) filters based on repeat analysis of a pooled QC sample. Authenticated serum lipids were quantified using a class-specific lipid internal standard for data normalization.

### Statistical analysis

Descriptive statistics were assessed using a Wilcoxon test and a Fisher’s exact test. Complementary multivariate data analysis techniques were used to identify time-dependent changes among 197 annotated serum metabolites and lipid species associated with the PD intervention using MetaboAnalyst 6.0 (Pang et al., 2024), including unsupervised principal component analysis (PCA) and hierarchical cluster analysis (HCA), as well as supervised orthogonal partial least-squares-discriminate analysis (OPLS-DA), and volcano plots. In the latter case, serum metabolites/lipids were deemed significant if they exceeded a fold-change (FC > 1.3) and had a p-value (*p* < 0.05) after a false-discovery rate (FDR) or Bonferroni adjustment for multiple hypothesis testing. In addition, top-ranked serum metabolites/lipids and their ratios were further classified based on receiver operating characteristic (ROC) curves and an analysis of variance (ANOVA) when comparing participants in the treatment arm (n = 37) relative to the control (n = 5) with paired serum samples collected at baseline and after 8 weeks. In most cases, serum metabolomic data were *log*_*10*_-transformed and autoscaled prior to multivariate data analysis, with missing values inputted using a k-nearest neighbor (feature wise) algorithm unless otherwise noted. A pathway analysis using MetaboAnalyst 6.0 was also performed on serum metabolomic data for a subset of metabolites/lipid species having accessible HMDB ID entries (84 of 197) to verify specific metabolic pathways impacted in study participants after 8 weeks of PD relative to baseline after an FDR adjustment. For parameter settings in the pathway analysis, which used a scatter plot as the visualization method, a global test was selected as the enrichment method and a relative-between centrality as the topology measure, using all compounds from the KEGG pathway for *Homo sapiens* following *log*_*10*_-transformation and autoscaling of data.

A multi-omics classification method was used to integrate 12 top-ranked serum metabolites/lipids, 470 methylation sites, and 9 cognitive skill measures to identify discriminant features for participants from baseline (pre) and following PD meditation (post). In this case, Data Integration Analysis for Biomarker discovery using Latent Components (DIABLO) (Singh et al., 2019) using the mixOmics R Bioconductor package (Rohart et al., 2017) was applied as a supervised learning technique that selects the most discriminant variables from multiple datasets to describe a categorical outcome variable, in this case pre- from post-PD intervention. The model is made of components that are sums of differently weighted variables from each dataset. Datasets were transformed using the natural logarithm and then centered and scaled using the *scale* function in R. As the study design included repeated measures from the same individual, a multilevel approach was used to isolate the within-sample variation from each dataset; then, DIABLO modeling was performed.

To explore the correlation among the datasets, regression analysis with PLS was run, which led to the choice of a design matrix with a weight of 0.5 as the cross-correlations ranged between 0.5 and 0.7. To select the number of components in the model, *block.splsda* function was run with 5-fold cross-validation and 100 repeats. One component was selected by using the centroid distance for an error rate of 4%. Next, the *tune.block.splsda* function was used to choose the optimal number of variables from each block, which was 5 serum metabolites/lipids, 25 methylation sites, and 7 cognitive skills. To visualize the data, *plotDiablo* was run to demonstrate the overall correlation between the selected variables of the datasets, *plotLoadings* was run to demonstrate the weights of the selected variables, *circosPlot* was run to demonstrate the correlations >0.5 between the datasets, and *cimDiablo* heatmap was run to demonstrate the signature expression for each variable for each sample. Model performance was tested with 5-fold cross-validation repeated 10 times. ROC curves were generated to show the performance of each data block.

## Results

### Study participant demographics

All participants were students at FIU and had no prior experience with meditation at the onset of the study. The median age of the treatment group was 23.5 years (IQR 22–26.8), while the median age for the control group was 22 years (IQR 21–23), which were not statistically significant from each other (p = 0.4236). Twenty-nine participants in the treatment group were women (76.3%), while only one woman was in the control group (20%). Gender imbalance is a noted limitation of this study.

### Untargeted metabolomic data analyses: serum biomarkers of PD intervention

Untargeted metabolomics analysis was conducted on paired serum samples collected from 38 healthy university students at baseline and 8 weeks following PD to identify metabolic phenotype changes associated with this meditation intervention. Overall, a total of 197 annotated compounds in serum (66 metabolites and 131 lipid species) were reliably measured with adequate frequency and technical precision after blank subtraction using a cross-platform strategy and standardized data workflow for untargeted metabolite analysis. MSI-CE-MS was used for the rapid analysis of polar/hydrophilic metabolites from serum filtrates under positive and negative ion modes, whereas reversed-phase (RP) LC-MS provided an orthogonal platform for the analysis of six major lipid classes from serum ether extracts under the negative ion mode. In all cases, most serum metabolites were identified based on spiking with authentic standards (except 20 unknown ions), whereas all lipids were annotated based on their sum notation with consistent retention time trends within a homologous lipid group, including 44 phosphatidylcholines (PCs), 25 fatty acids (FAs), 22 phosphatidylethanolamines (PEs), 20 sphingomyelins (SMs), 11 phosphatidylinositols (PIs), and 10 ceramides (Supplementary Figure S1).

**FIGURE 1:**
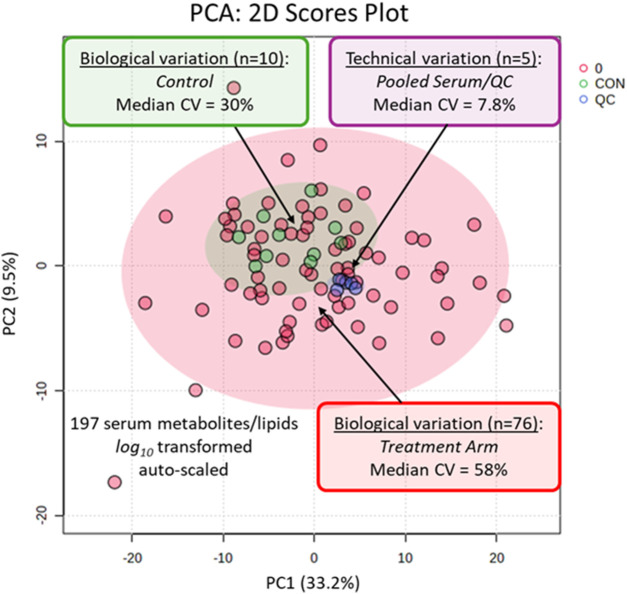
A PCA depicting the data variance in a 2D score plot based on 197 serum metabolites and lipids measured consistently from 38 paired participants at baseline and after 8 weeks of PD (n = 76) as compared to five paired participants as controls (n = 10), as well as repeat analysis of pooled QC samples (n = 5). Overall, there was good technical precision (median CV = 7.8%) as compared to the larger biological variance in both study participants (median CV = 58%) and controls (median CV = 30%).

**FIGURE 2:**
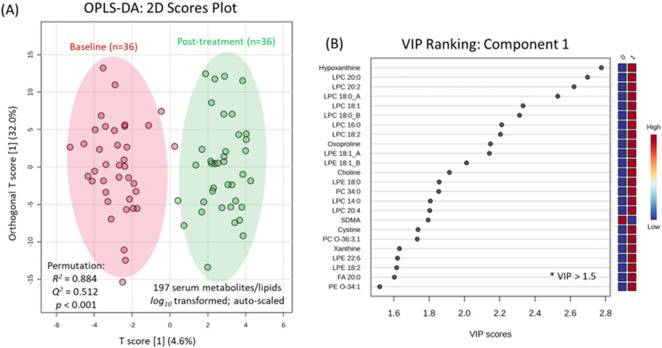
**(A)** A 2D score plot when using OPLS-DA was used to characterize temporal changes in the serum metabolome in paired serum samples from study participants collected at baseline and following 8 weeks of PD. A permutation test with 1,000 iterations was performed to verify a robust model, as reflected by *R* ^*2*^ = 0.884, *Q* ^*2*^ = 0.512, *p* < 0.001. **(B)** A VIP plot for component 1 was used to identify top-ranked serum compounds associated with the PD intervention (VIP score >1.5), including 6 metabolites and 18 lipids (primarily LPCs and LPE species).

Overall, acceptable technical precision was achieved based on repeat analysis of pooled QC samples (n = 5) with a median coefficient of variance (CV) of 7.8% measured for 197 serum metabolites/lipids relative to the larger biological variation in the serum metabolome from all study participants (median CV = 58%, n = 76) and controls (median CV = 30%, n = 10), as shown in the PCA 2D score plot (Figure 1). Representative recovery standards measured in all serum and QC samples through MSI-CE-MS and RP-LC-MS are also demonstrated in the control charts, showing adequate intermediate precision with a mean CV < 17% (Supplementary Figure S2). We next performed a supervised multivariate data analysis by OPLS-DA to identify putative treatment response biomarkers temporally associated with the PD intervention. A 2D score plot demonstrated excellent differentiation of study participants based on changes in their serum metabolomic profiles following 8 weeks of PD as compared to baseline with good model performance after a permutation test with a *Q* ^*2*^ = 0.512 (Figure 2A). A variable importance in projection (VIP) plot along component 1 was used to rank order serum metabolites/lipids (Figure 2B) that, in most cases, increased in concentration following the PD meditation intervention using a minimum cut-off (VIP >1.5). Compounds with high scores in the VIP plot included hypoxanthine, oxoproline (pyroglutamic acid), symmetric dimethylarginine (SDMA), cystine, and xanthine, along with a series of lysophosphatidylcholines (LPCs) and lysophosphatidylethanolamines (LPEs). Similarly, a volcano plot using a paired t-test (p < 0.05, FDR adjustment) with a minimum fold-change (FC > 1.3) cut-off identified 20 serum metabolites/lipids that increased in circulation after 8 weeks of PD from baseline (Supplementary Figure S3), which was consistent to the results observed with the OPLS-DA model.

**FIGURE 3:**
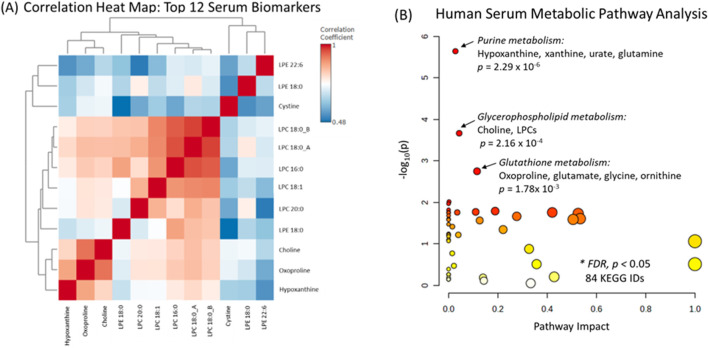
**(A)** A Pearson correlation matrix for the 12 top-ranked serum metabolites and lipids that undergo changes in their levels after 8 weeks of PD relative to baseline that satisfied a Bonferroni correction (*p* < 2.54 × 10^−4^) after autoscaling and *log*-transformation. Overall, three distinct clusters of co-linear metabolites/lipids in serum were evident, including a series of LPC species (LPC 16:0, LPC 18:0 isomers etc.), hypoxanthine–oxoproline–choline, and cystine–LPE species. **(B)** A metabolic pathway analysis of global serum metabolomic changes among 84 metabolites/lipids with KEGG IDs in 38 study participants following 8 weeks of PD intervention relative to baseline. Overall, three metabolic pathways associated with purine degradation, glycerophospholipid metabolism, and glutathione biosynthesis satisfied a false discovery (FDR) adjustment for significance.

**FIGURE 4:**
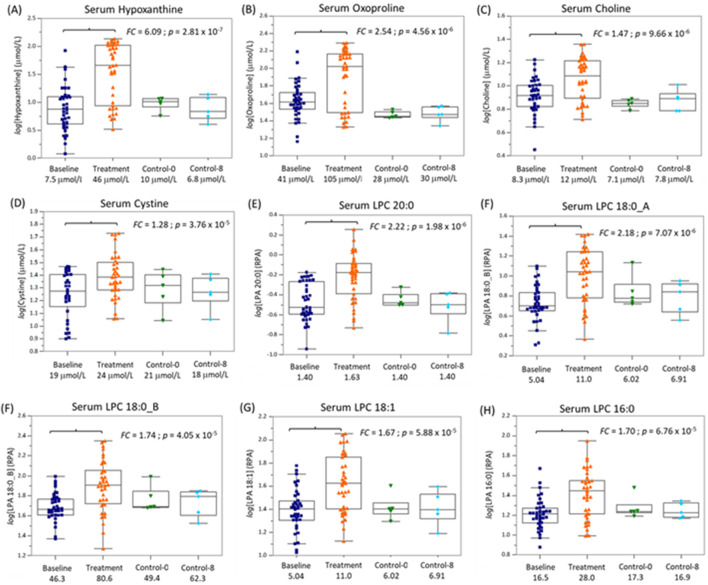
Box plots and ANOVA for nine (of the 12) top-ranked serum metabolites/lipid species that increased specifically in study participants (n = 38) following the PD intervention relative to controls at two time points (baseline; 8 weeks). In this case, hypoxanthine **(A)**, oxoproline **(B)**, choline **(C)**, cystine **(D)**, as well as five LPC species, **(E)** LPC 20:0, **(F)** LPC 18:0_A, **(G)** LPC 18:0_B, **(H)** LPC 18:1, **(I)** LPC 16:0, which were found to increase significantly (*p* < 2.54 × 10^−4^, Bonferroni adjustment) in study participants unlike controls who did not partake in PD. Median serum metabolites are reported in absolute concentrations (μmole/L), whereas LPCs are reported as ion responses (i.e., relative peak area) after normalization to LPC-18:1-d7.

A stringent Bonferroni correction (*p* < 2.45 × 10^−4^) was then applied to this panel of serum metabolites to minimize the risk for potential false discoveries. In this case, the concentrations of 12 metabolites/lipids, namely, hypoxanthine, oxoproline, choline, cystine, LPE 18:0, LPE 18:1_A, LPE 22:6, LPC 16, LPC 18:0_A, LPC 18:0_B, LPC 18:1, and LPC 20:0, were found to significantly increase after PD intervention (Table 1). Furthermore, these lead biomarker candidates were measured with good technical precision in pooled QC serum samples with a mean CV of 6.8% (n = 8) and 11.5% (n = 5) for serum metabolites and lipids, respectively. A Pearson correlation matrix analysis was next performed to further explore the relationship among the 12 top-ranked serum metabolites and lipids among study participants (Figure 3A). Three distinct clusters of circulating metabolites/lipids were found to be highly co-linear, namely, choline, oxoproline, and hypoxanthine (*r* = 0.801–0.923), LPC species (*r* = 0.849–0.902), along with cystine and LPE species (*r* = 0.520–0.555) Next, an enrichment analysis was performed to characterize global metabolic pathways likely modulated by the PD intervention that satisfied false discovery rate (FDR) adjustment (Figure 3B). This analysis validated the top-ranked serum metabolites and lipids identified via the OPLS-DA model, paired t-test, and correlation matrix, highlighting three key metabolic pathways, namely, purine metabolism (e.g., hypoxanthine and xanthine), glycerophosphate metabolism (e.g., LPC species), and glutathione metabolism (e.g., oxoproline and cystine). In fact, serum hypoxanthine and LPC 20:0 were the two top-ranked treatment response biomarkers that differentiated study participants after PD as compared to the baseline, as assessed by ROC curves, with area under the curve (AUC) values of 0.821 (*p* = 4.88 × 10^−8^) and 0.808 (*p* = 1.72 × 10^−6^), respectively (Supplementary Figures S4A,B). Improved discrimination was achieved for PD study participants when using a ratiometric biomarker in the ROC curves, including serum hypoxanthine:lysine or hypoxanthine:acetylcarntine ratio (AUC = 0.860, *p* < 1 × 10^−9^) (Supplementary Figures S4C,D). Lastly, a series of box plots after an ANOVA illustrate a selective increase in lead serum biomarker candidates in the study group (with PD) after 8 weeks of PD compared to baseline and controls (i.e., no PD intervention). Importantly, no significant changes were observed in the levels of these 12 metabolites/lipids in the control group after 8 weeks compared to baseline. In contrast, the study group exhibited a significant increase in these metabolites after 8 weeks of PD, relative to both their baseline and the control group, suggesting that this selective increase is attributable to the PD intervention rather than other factors (Figure 4). In this case, median concentrations (micromole/L) or relative ion responses (RPAs) are reported for four serum metabolites (hypoxanthine, oxoproline, choline, and cystine) and five LPC species (e.g., two LPC 18:0 isomers, LPC 18:1, LPC 16:0, and LPC 20:0), respectively, highlighting an increase in the response following PD from baseline over an 8-week period, which was not observed among controls. Overall, there was considerable between-subject heterogeneity in serum metabolite and lipid changes, particularly in hypoxanthine and oxoproline, which may reflect differences in adherence to the practice or individual responses to the PD intervention.

**TABLE 1:** Summary of 12 leading serum metabolites/lipids that increased after an eight-week PD meditation intervention from baseline for 38 participants that satisfied a Bonferroni correction.

Compound^a^	HMDB#	Median FC^b^	p-value^b^
Hypoxanthine	HMDB0000157	2.55	2.81 × 10^−7^
LPC 20:0	HMDB0010390	1.77	1.98 × 10^−6^
Oxoproline (pyroglutamic acid)	HMDB0000267	1.88	4.56 × 10^−6^
LPC 18:0_A^c^	HMDB0011128	1.90	7.07 × 10^−6^
LPE 22:6	HMDB0011526	1.47	8.64 × 10^−6^
Choline	HMDB0000097	1.44	9.66 × 10^−6^
LPE 18:1_A^c^	HMDB0011505	1.48	1.67 × 10^−5^
LPC 18:1	HMDB0002815	1.56	3.21 × 10^−5^
LPC 16:0	HMDB0010382	1.49	3.28 × 10^−5^
Cystine	HMDB0000192	1.36	3.76 × 10^−5^
LPC 18:0_B^c^	HMDB0010384	1.41	4.05 × 10^−5^
LPE 18:0	HMDB0011130	1.44	1.10 × 10^−4^

^a^ Metabolites were identified with high confidence (level 1) after spiking with authentic standards, whereas lipids were annotated based on their sum notation and retention time relationships within a homologous series (level 2).

^b^ A paired t-test on log-transformed serum metabolites/lipids was used for statistical data analysis with median fold change (FC)-based paired changes in responses after PD meditation relative to baseline.

^c^ Chromatographically resolved lipid positional isomers were annotated based on their retention time order (A, B, etc.) and likely reflect their likely fatty acyl chain on the sn-1 or sn-2 position.

# denotes number.

### DIABLO analysis integrating metabolites, methylation sites, and cognitive skills

**FIGURE 5:**
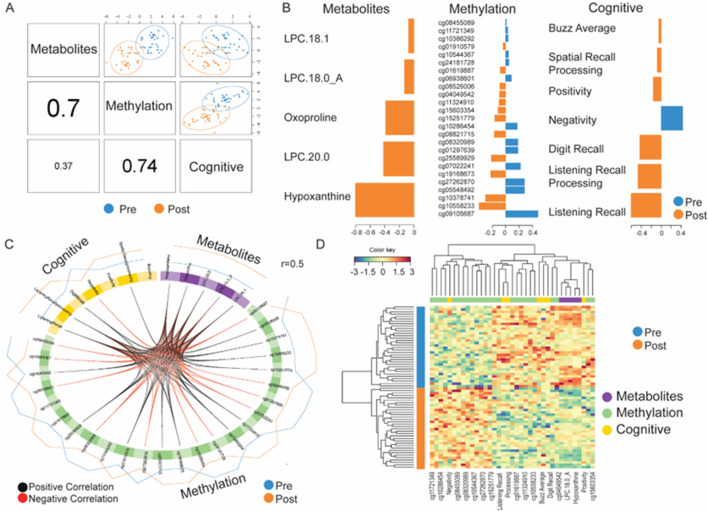
DIABLO integrative multi-omic analyses of metabolites, DNA methylation sites, and cognitive skill measures create a model to discriminate participants at baseline (pre) from those after 8 weeks of PD meditation intervention (post). **(A)** The correlation matrix shows the separation of paired participants before and after PD meditation based on the first component of the model between datasets. **(B)** Loading weights of the most significant variables in the first component of the model. **(C)** Circos plot showing a correlation greater than 0.5 between the most discriminatory variables. Black lines denote positive correlations, and red lines denote negative correlations. Around the perimeter, orange lines denote the level in the post-condition and blue lines denote the level in the pre-condition. **(D)** The clustered image map represents the DNA methylation, metabolite, and cognitive skill variable for each participant in the study at baseline and after PD intervention.

To further understand the mechanistic role of these PD meditation treatment response biomarker candidates, we performed DIABLO analysis to integrate these 12 most discriminating serum metabolites/lipid species and the previously determined 470 differentially expressed methylation sites, along with nine cognitive skills. The overall Pearson correlation coefficient (r) between DNA methylation and cognitive skills was 0.74, that between serum metabolites/lipids and DNA methylation sites was 0.70, and that between serum metabolites/lipids and cognitive skills was 0.37 (Figure 5A). The developed model had 1 component with 5 serum metabolites/lipids, 25 DNA methylation sites, and 7 cognitive skills. These five circulating compounds were identified as hypoxanthine, oxoproline, LPC 18:1, LPC 18:0_A, and LPC 20:0. The most highly weighted serum metabolite was hypoxanthine (−0.805), the DNA methylation site was *cg09105687* (0.484), and cognitive skill was listening recall (−0.607) (see Figure 5B for full loading weights). The absolute value of the loading weight indicates its importance, as visualized by the length of the bar in Figure 5B. A negative loading weight indicates higher values in the post-condition, visualized in orange, while positive values indicate higher values in the pre-condition, visualized in blue. A circos plot shows the correlation between these three multi-omics datasets, with the Pearson correlation coefficient r > 0.50. Positive correlations are in black, negative correlations are in red, and the expression level of each component is around the perimeter with pre-levels in blue and post-levels in orange. All the serum metabolites/lipids increase in concentration following PD meditation. Digit recall, listening recall, and listening recall processing showed strong positive correlations with all five serum metabolites/lipids, suggesting a link between memory performance and metabolic adaptive responses to the PD intervention. Additionally, spatial recall processing also had a positive correlation with serum hypoxanthine and oxoproline. Interestingly, the negativity score had a negative correlation with hypoxanthine, oxoproline, and LPC 18.0_A.

Correlations were also observed between the differential DNA methylation sites and serum metabolites/lipids. A set of nine DNA methylated sites had a positive correlation with all five metabolites/lipids (*cg19168673*, *cg15251779*, *cg10558233*, *cg11324910*, *cg04049542*, *cg15603354*, *cg10378741*, *cg01910579*, and *cg25589929*), while another set of nine differentially DNA methylated sites had a negative correlation with all five metabolites/lipids (*cg09105687*, *cg24181728*, *cg07022241*, *cg08320989*, *cg08455089*, *cg01297639*, *cg10544367*, *cg06938601*, and *cg27262870*). For correlations between methylation sites and cognitive skills, *cg19168673* and *cg25589929* had a positive correlation with digit recall, listening recall, and listening recall processing. Additionally, listening recall and listening recall processing had a positive correlation with six DNA methylated sites (*cg10558233*, *cg11324910*, *cg04049542*, *cg15603354*, *cg10378741*, and *cg01910579*) and had a negative correlation with five DNA methylated sites (*cg07022241*, *cg08320989*, *cg01297639*, *cg10544367*, and *cg27262870*) (Figure 5C). The molecular signature for pre vs. post is displayed in the heatmap (Figure 5D). Model performance was tested using 5-fold cross-validation repeated 10 times, with an overall AUC of 0.956. For each block individually, the AUC was 0.932 for metabolites, 1 for methylation, and 0.938 for cognitive skills (Supplementary Figure S5). Supplementary Table S1 includes the list of DNA methylated sites showing a correlation greater than 0.50. This table summarizes the chromosome number, strand location, if the gene is part of an island, relation of the methylation site in proximity of the island, gene name, and level of methylation (beta value differences).

## Discussion

In this study, we conducted a cross-platform metabolomic study on serum samples collected from 38 healthy and novice-to-meditation college students following an 8-week PD meditation course. Complementary multivariate and univariate statistical data analysis methods were applied to identify circulating metabolites and lipids that increased following the PD intervention from baseline for participants (and controls) while satisfying a Bonferroni correction (p < 7.2 × 10^−4^). This approach revealed 12 serum metabolites/lipids of significance, namely, hypoxanthine, oxoproline (or pyroglutamic acid), choline, and cystine, as well as three LPE and five LPC species that were measured using orthogonal MSI-CE-MS and reversed-phase LC-MS methods. Overall, there was considerable between-subject variation in serum biomarker responses to the intervention that, we speculate, may relate to the adherence, effort, and consistency of the practice, which warrants further study. An enrichment analysis confirmed that these PD-responsive metabolites/lipids were largely associated with three key metabolic pathways, namely, purine degradation cycle, glutathione biosynthesis, and glycerophospholipid metabolism that were also co-linear with each other. Purine metabolism plays a vital role in maintaining cellular energy balance, and hypoxanthine is elevated in the bloodstream following strenuous exercise and skeletal muscle contractions due to irreversible ATP breakdown (Kuehnbaum et al., 2015). However, normally approximately 90% of hypoxanthine is reutilized and converted into inosine monophosphate through the salvage pathway of purine metabolism, which recycles the fundamental components for the reconstitution of DNA, RNA, and ATP (Saugstad, 1988; Murray, 1971). As PD meditation includes some yogic exercises, an increase in hypoxanthine following the PD intervention from baseline may reflect this process in contrast to other meditation methods that do not involve physical activity. As purine metabolism plays a vital role in maintaining cellular energy balance, our results suggest that meditation may help in maintaining energy balance while regulating oxidative stress since hydrogen peroxide is a redox-dependent signaling molecule and by-product of xanthine oxidase activity (Kelley et al., 2010). Interestingly, both xanthine and notably hypoxanthine levels were elevated following PD meditation in our study, although the increase in serum xanthine levels did not satisfy a Bonferroni correction. A recent study reported that hypoxanthine may also elicit antidepression effects in patients with higher levels, resulting in suppression of systemic and hippocampal inflammation that can alleviate chronic unpredictable mild stress and social defeat stress in a mouse model (Wu et al., 2024). As a result, increased hypoxanthine levels in the post-meditation group, observed in our study, may suggest the involvement of hypoxanthine in mediating cognitive improvements in memory recall and listening. However, our study did not examine changes in mental health conditions of participants, including depression and anxiety.

Another important emerging role of hypoxanthine is in maintaining intestinal barrier function. Using human T84 intestinal epithelial cells, Lee et al. (2018) demonstrated that hypoxanthine modulates energy metabolism in intestinal epithelial cells and is critical for maintaining intestinal barrier function. This is highly relevant in gastrointestinal diseases such as irritable bowel syndrome (IBS) and inflammatory bowel disease (IBD), where impaired intestinal epithelial barrier function is a hallmark in the development and perpetuation of these conditions (Sommer et al., 2021). In a chemically induced model of colitis, it was shown that loss of hypoxanthine in the epithelium correlated with disease severity and that supplementation of this metabolite or selective colonization with purine-producing bacteria improved cellular energetics while promoting mucus generation and epithelial barrier function, resulting in enhanced wound healing and protection from colitis. It has been proposed that purines, such as microbiome-derived hypoxanthine, facilitate efficient nucleotide biosynthesis and that intestinal epithelial cells (IECs) may enhance their energy balance by preferentially salvaging exogenous purines for ATP production (Lee et al., 2018; Lee et al., 2020). A recent study showed reduced levels of hypoxanthine in stool samples of patients with IBS-C and IBS-D, which could reflect decreased production or elevated breakdown of hypoxanthine by the gut microbiome of IBS patients (Mars et al., 2020). Our study showed an increase in the levels of hypoxanthine following an 8-week meditation course, suggesting that PD could be a beneficial integrative therapy in managing patients with such gastrointestinal diseases (Ge et al., 2022). Previous studies on meditation and yoga have not reported changes in this metabolite; however, in our study, we observed that PD meditation modulated hypoxanthine levels. This discrepancy may reflect differences in the analytical techniques used or the distinct practices involved in various forms of meditation and yoga.

Choline, an essential nutrient for humans, serves as the precursor for phosphatidylcholine and acetylcholine, which are essential for maintaining cellular membrane integrity and supporting neural function. Acetylcholine is an important neurotransmitter for memory, mood, muscle control, and other brain and nervous system functions (Zeisel and da Costa, 2009). Furthermore, as a methyl donor and cofactor, it plays a major role in DNA synthesis and methylation, cell division, and detoxification. Additionally, deficiency of choline is associated with cardiovascular diseases, IBS, IBD, non-alcoholic fatty liver disease, chronic kidney disease, and gut microbiota dysbiosis (Arias et al., 2020). Similar to hypoxanthine, we observed an increase in serum concentrations of choline following 8 weeks of PD in participants who were requested to maintain their habitual diet and physical activity throughout the study. Considering the above-mentioned beneficial effects of choline on human metabolic processes, these findings strongly suggest that PD meditation positively influences both the mind and body by modulating choline metabolism.

Oxoproline and cystine are polar metabolites involved in glutathione metabolism. Cystine, an abundant and stable oxidized thiol disulfide in circulation, is imported within the cells and is reduced to cysteine, which serves as a key limiting reagent needed for glutathione biosynthesis. Glutathione plays a critical role in protecting cells from oxidative stress and is essential for supporting immune function and detoxification of oxidants, carcinogens, and other intoxicants (Silvagno et al., 2020; Kennedy et al., 2020). In our study, PD resulted in an increase in concentration for both cystine and oxoproline in circulation, suggesting that this intervention may help in reducing cellular oxidative stress by modulating glutathione synthesis. It is important to note that, indeed, an enrichment analysis revealed glutathione metabolism as a significantly modulated pathway in response to PD. Oxoproline (or pyroglutamic acid) is derived from the cyclization of glutamate that is involved in glutathione biosynthesis and recycling and thus is a measure of glutathione turnover (Bachhawat and Yadav, 2018). Although oxoprolinemia has long been associated with glutathione synthetase deficiency, metabolic acidosis, and acetaminophen toxicity (Emmett, 2014), oxoproline has also been reported to mediate potential health benefits relevant to cognition and memory function (Grioli et al., 1990; Yang et al., 2021). For example, a Mendelian randomization study revealed that serum oxoproline was causally associated with higher scores in different neurocognitive tests and general intelligence, as mediated by 25 single-nucleotide polymorphisms as instrument variants (Saugstad, 1988). Our multi-omics analysis revealed similar beneficial effects of oxoproline, showing a positive correlation with listening recall, digit recall, and listening recall processing. Importantly, serum oxoproline, choline, and hypoxanthine levels were highly co-linear, suggesting a common underlying metabolic phenotype in response to PD meditation.

LPCs were originally hypothesized to be proinflammatory lipid mediators; however, recent studies have found that lower plasma LPC levels are associated with unfavorable disease outcomes (Knuplez and Marsche, 2020). Decreased levels of LPCs were reported in rheumatoid arthritis (Fuchs et al., 2005), diabetes (Barber et al., 2012), Alzheimer’s disease (Mulder et al., 2003; Semba, 2020; Zha et al., 2025), schizophrenia (Wang et al., 2019), and pulmonary arterial hypertension (Chen et al., 2020). Furthermore, recently, it was reported that microbiota-derived LPC significantly reduced Alzheimer’s disease pathology and improved cognitive impairment (Zha et al., 2025). In our study, the levels of six LPC species increased following PD relative to baseline and controls, including two LPC 18:0 isomers (denoted A and B) resolved via chromatography, along with LPC 18:1, LPC 16:0, and LPC 20:0. Moreover, it was found that LPC 18:0, LPC 18:1, and LPC 20:0 positively correlated to three cognitive skills, namely, listening recall, listening recall processing, and digital recall. Interestingly, ROC curve analysis revealed that serum hypoxanthine, LPC20:0, hypoxanthine:lysine ratio, and hypoxanthine:acetylcarnitine ratio have the potential to serve as treatment response biomarkers when evaluating the effectiveness of PD practices (Supplementary Figure S4).

Meditation protocols and practices differ widely, which makes a generalizable biomarker signature at the genetic, epigenetic, and metabolomic levels a challenge. However, Antonelli et al. (2024) recently summarized the effects of different meditation studies on human blood lipid levels, especially blood cholesterol and triglycerides. Additionally, another recent study on brief meditation intervention revealed a similar impact of meditation on amino acid and lipid metabolism (Tang et al., 2025). Previously, our group revealed the impact of PD on the cognitive skills, gene expression pattern, and DNA methylation (Pragya et al., 2021; Abomoelak et al., 2022; Pragya et al., 2023; Abomoelak et al., 2023). In this study, we applied, for the first time, a multi-omics approach with a stringent cutoff (r = 0.5) to identify potential correlations among cognitive skills, DNA methylation pattern, and the 12 significantly altered serum metabolite and lipid signatures. The list of DNA methylation sites correlating with the cognitive skills and metabolites belonged to essential genes and regulators known to be involved in cellular functions, signal transductions, and metabolic disorders.

Digit recall, listening recall, and listening recall processing showed strong positive correlations with the five serum metabolites/lipids whose levels increased following PD meditation, including hypoxanthine, oxoproline, LPC 18:1, LPC 18:0_A, and LPC 20:0. Additionally, spatial recall processing also correlated positively with serum hypoxanthine and oxoproline, suggesting that PD can enhance alertness and memory performance, as reflected by adaptive metabolic phenotype changes. Interestingly, there was negative correlation of hypoxanthine, oxoproline, and LPC 18:0 with the negativity score, further supporting that these bioactive compounds may mediate the PD-induced beneficial effects on memory and alertness. Correlations were also observed between the differential DNA methylation sites and metabolites. Overall, nine differentially DNA methylated sites were positively correlated with five circulating metabolites, while another set of nine differentially DNA methylated sites were negatively correlated with all five serum metabolites, as illustrated in the circos plot. Most of these DNA methylation sites belonged to essential genes and regulators involved in signal transduction, synaptic regulations, cellular processes, growth, and differentiation. These results reveal the potential of PD meditation to reprogram key metabolic pathways, modulate DNA methylation, and positively impact participants’ cognitive outcomes.

The modest sample sizes for the intervention group (n = 38) and the control group (n = 5) are limitations of this study; however, the robust analysis techniques, stringent cutoff criteria, and the integration of multiomics datasets highlight the significance of the findings. Given the small sample size, there is a risk that the model may be overfitted, decreasing generalizability to larger samples; however, the model still provides insights into correlations between different data types and potential biomarkers for differentiating pre- from post-samples. In addition, a lack of diet tracking represents an additional limitation of this study since certain metabolites may derive, in part, from external sources (e.g., choline). However, we requested all participants to maintain their normal diet and lifestyle (e.g., physical activity) throughout the intervention period. In addition, as most recruited participants in our study were women (70%), a sex-stratified statistical analysis was not feasible due to inadequate study power. Indeed, Rojiani et al. (2017) reported that women may derive more psychological benefit (e.g., negative affect) than men in certain meditation or mindfulness interventions, which requires further investigation. This study supports the hypothesis that an 8-week PD meditation intervention influences cognitive functions at the molecular level by modulating DNA methylation, metabolite/lipid expression, and key metabolic pathways associated with purine degradation cycle, glutathione biosynthesis, and methylation/phospholipid metabolism. These changes contribute to the overall wellbeing observed after practicing PD meditation, with certain bioactive metabolites implicated in regulating inflammation, cognition, and intellectual performance. Assessment of changes to mental health outcomes and stress reduction following PD meditation is also warranted, given the impact on sleep quality, working memory, and the academic performance of students (Almarzouki et al., 2022).

## Conclusion

In summary, our study suggests a potential link between changes in serum hypoxanthine, oxoproline, and lysophosphatidylcholines, along with DNA methylation sites, associated with better cognitive outcomes of the participants following an 8-week PD meditation intervention. A larger multicenter study is still needed to improve study power while assessing the long-term physical and mental health and cognitive benefits of habitual PD meditation, as applied to diverse demographic groups.

## Data Availability

The raw data supporting the conclusions of this article will be made available by the authors, without undue reservation.
