# Supplementary material for: Preksha Dhyana meditation modulates the serum metabolome in healthy and meditation-naïve participants
Source: Front Mol Biosci. 2026 Apr 15;13:1741802. doi: 10.3389/fmolb.2026.1741802 (PMC13124489; doi:10.3389/fmolb.2026.1741802)
Supplement: Supplementary file 1 [file Supplementaryfile1.docx]

**SUPPLEMENTAL INFORMATION**


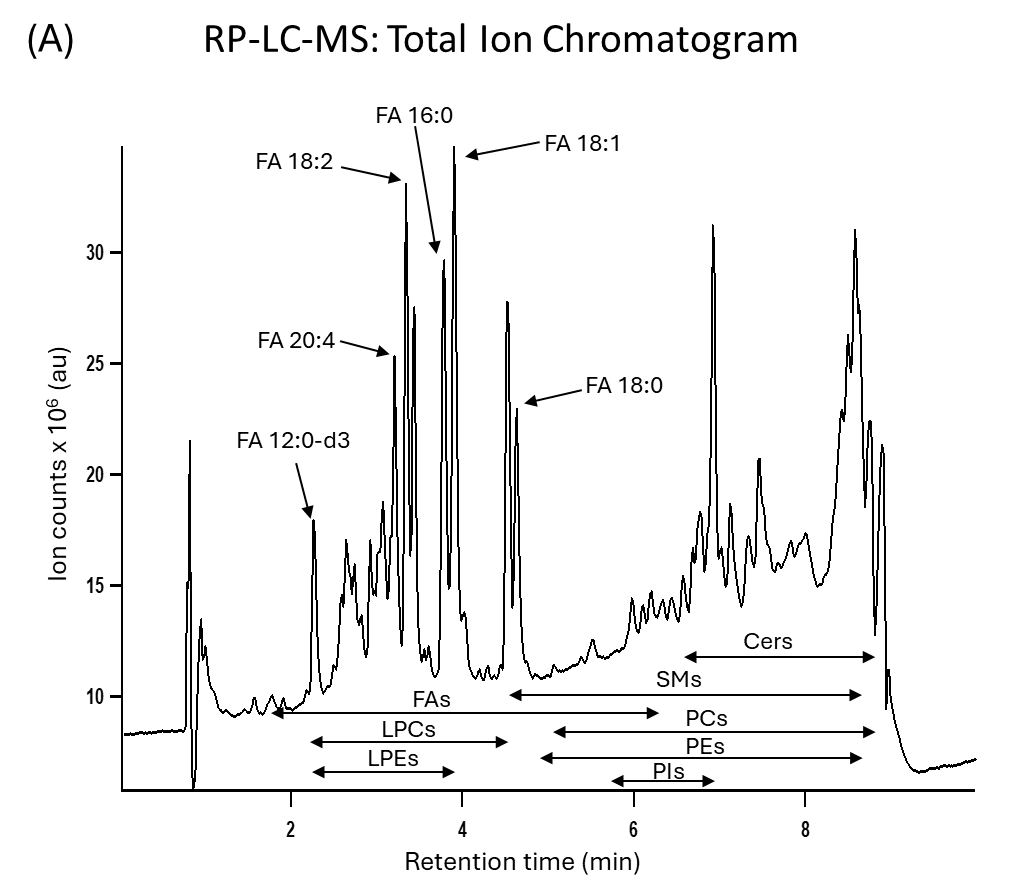


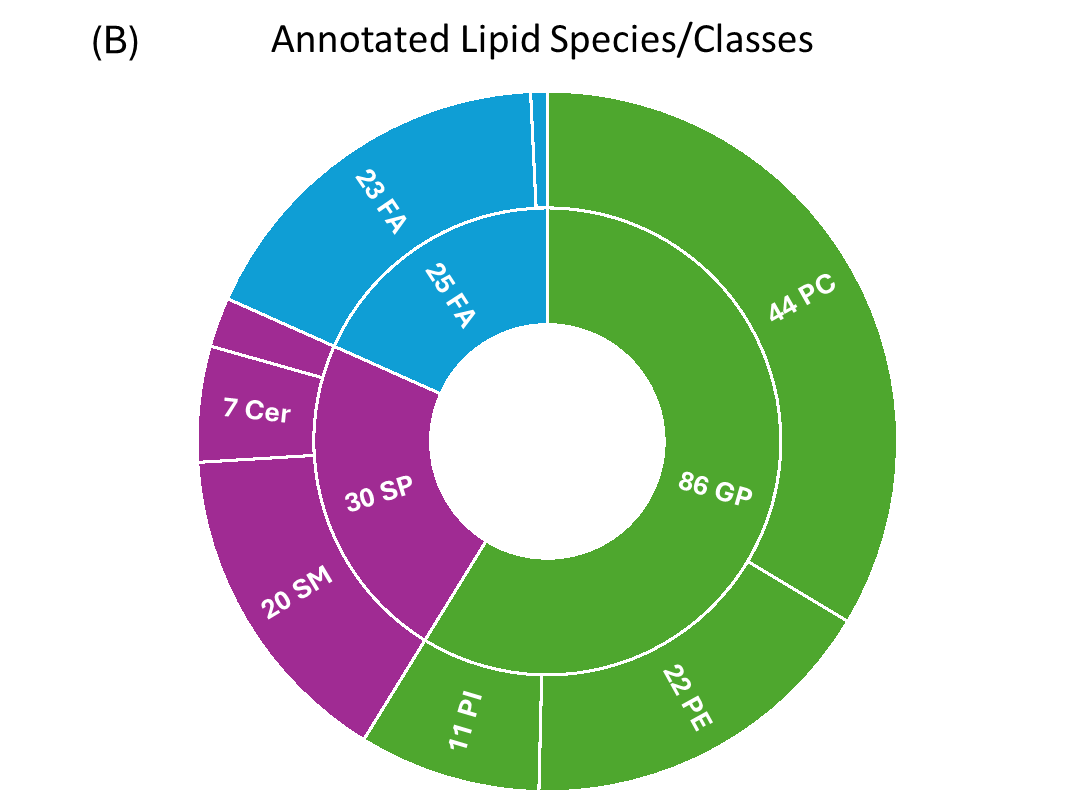


**Figure S1. (A)** Representative total ion chromatogram highlighting the elution order of major classes of lipids analyzed by RP-LC-MS under negative ion mode from serum ether extracts. **(B)** 131 serum lipids were annotated based on their sum notation and measured with adequate precision and frequency in this study, including 44 phosphatidylcholines (PCs), 25 fatty acids (FAs), 22 phosphatidylethanolamines (PEs), 20 sphingomyelins (SMs), 11 phosphatidylinositol (PIs) and 10 ceramides (Cers).


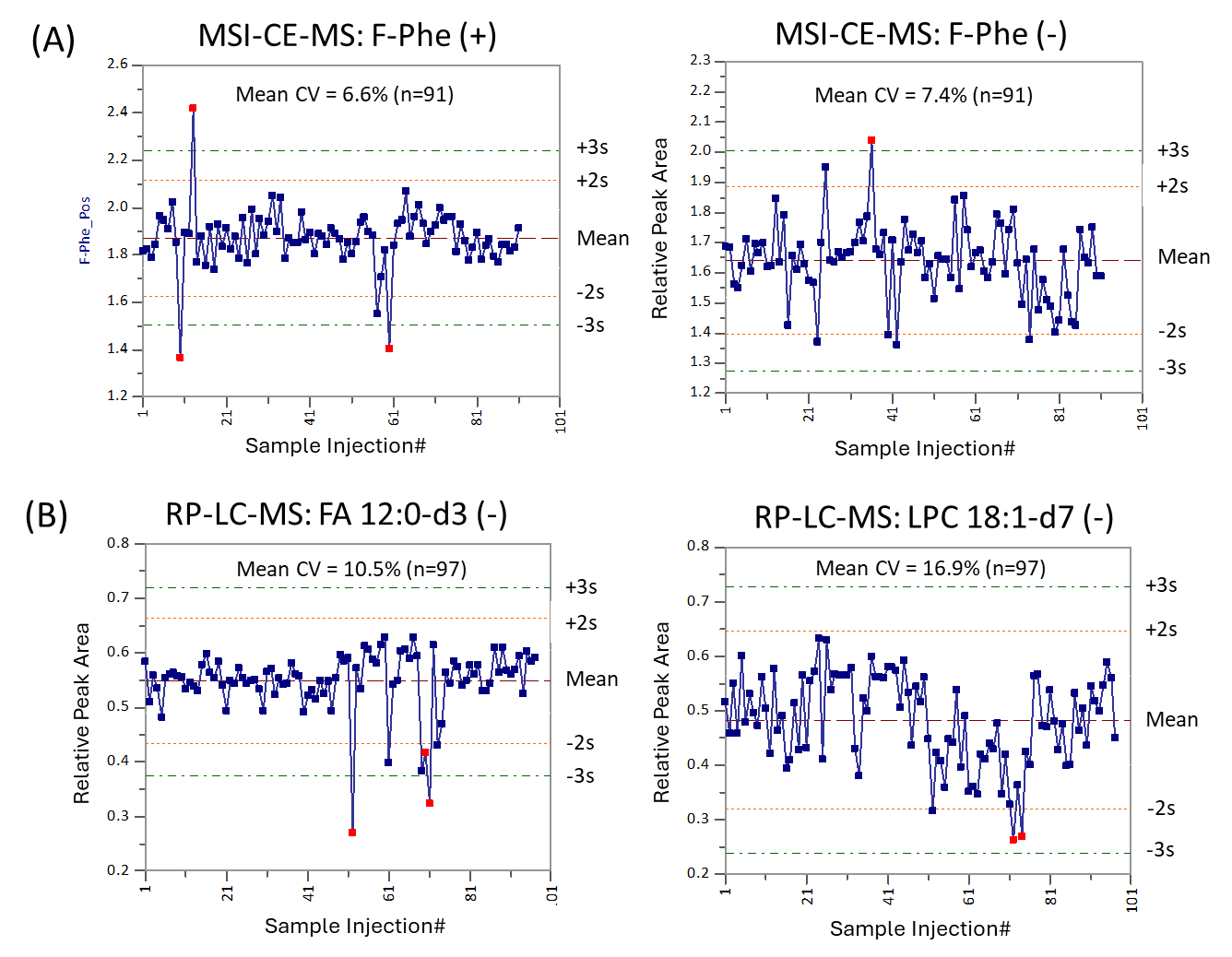


**Figure S2. (A)** Controls charts highlighting acceptable intermediate precision (mean CV < 8%) for F-Phe that was used as an internal standard in all serum samples and pooled QCs (every run) measured by MSI-CE-MS for cationic and anionic metabolites under positive and negative ion modes, respectively. **(B)** Control charts for two representative deuterated lipid internal standards highlighting acceptable intermediate precision (mean CV < 17% with outlier data < 5%) measured in all serum samples and pooled QC samples (every block of 10 runs) measured by RP-LC-MS for lipids under negative ion mode.


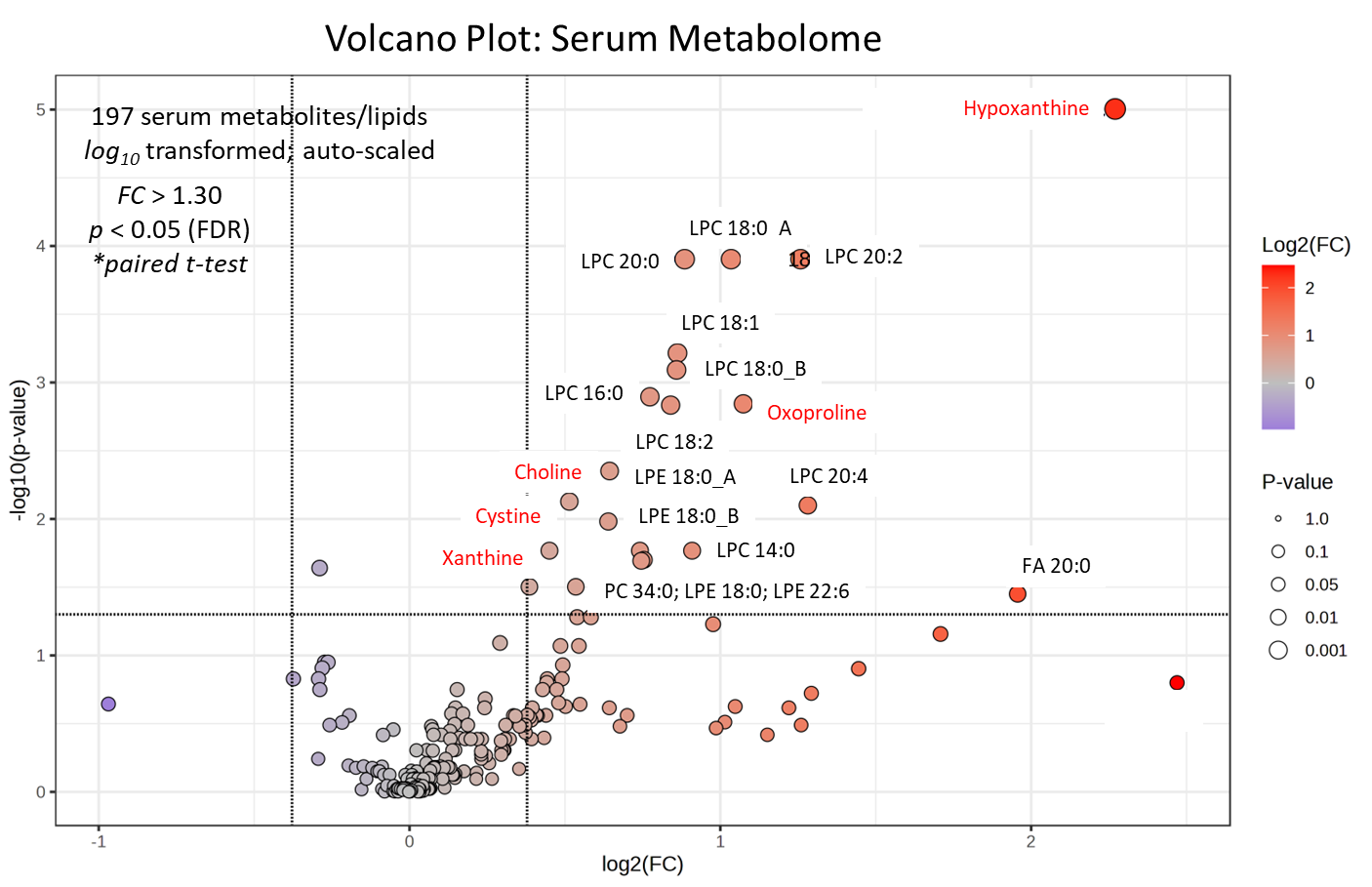


**Figure S3.** A volcano plot using a mean fold-change cut-off (FC > 1.3) and an adjusted p-value using a paired t-test (p < 0.05, FDR) similarly identified 5 metabolites and 15 lipid species as significantly elevated in circulation after 8 weeks of PD relative to baseline. These results were largely consistent with independent supervised multivariate data analysis using OPLS-DA and a global metabolic pathway analysis.


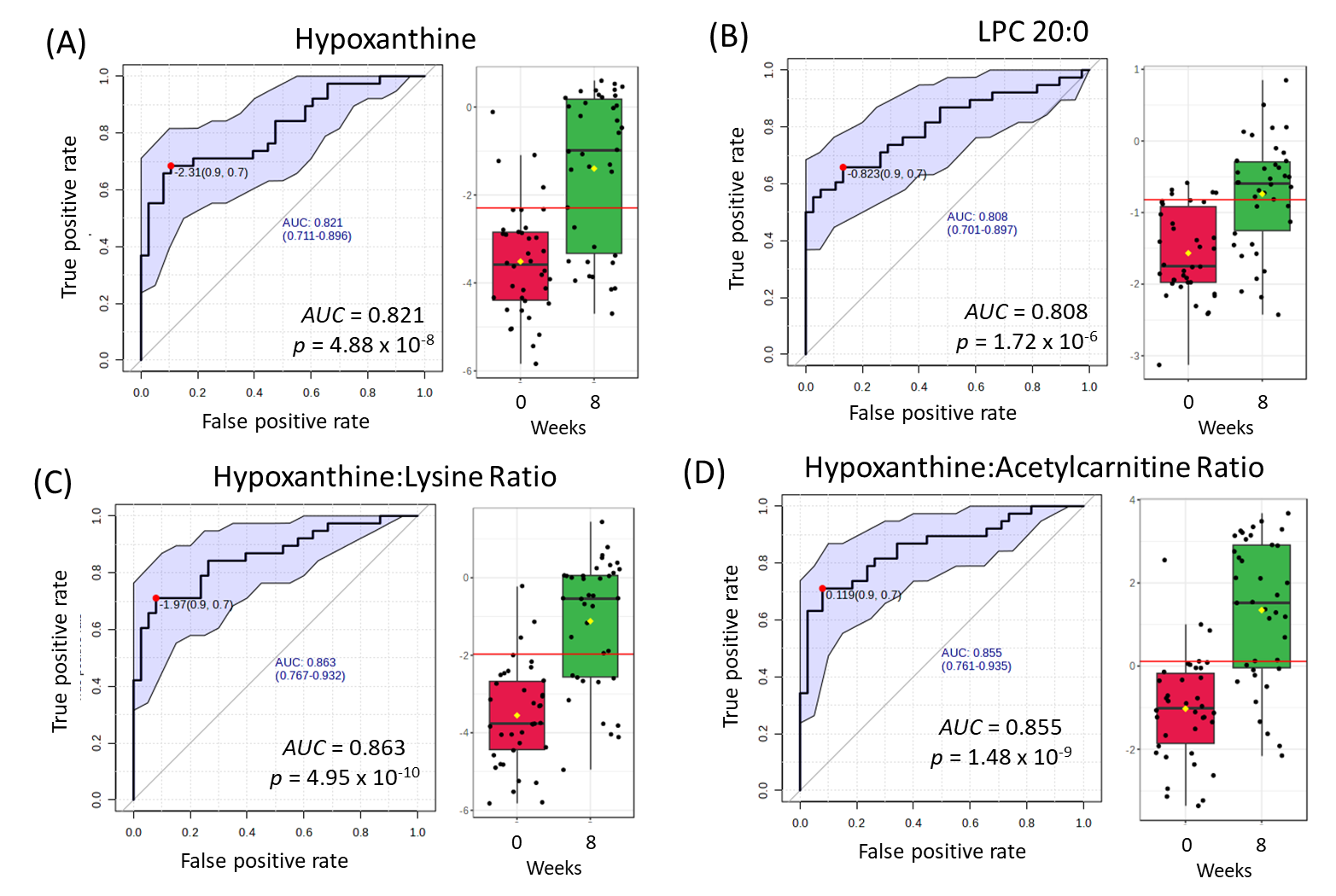


**Figure S4.** Series of receiver operating characteristic (ROC) curves for single and ratiometric serum biomarkers the increase in circulation after 8 weeks of PD intervention in study participants (n=38) relative to baseline levels (0 weeks), including **(A)** hypoxanthine, **(B)** LPC 20:0, **(C)** hypoxanthine:lysine ratio, and **(D)** hypoxanthine:acetylcarnitine ratio.


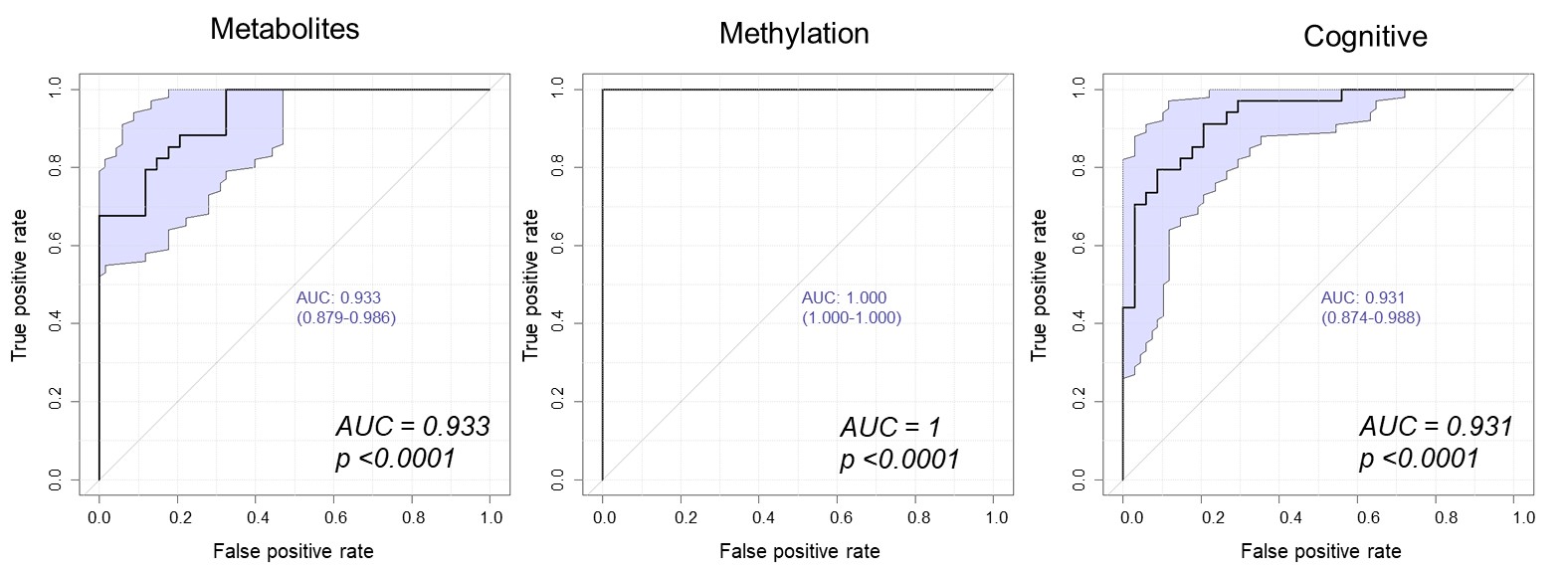


**Figure S5**. ROC curves with 95% CI in grey depict the model performance for each block of the DIABLO model with 5 M-fold cross validation repeated 10 times.

**Table S1.** The list of identified DNA methylations sites from DIABLO analysis. The corresponding chromosome, position on positive or negative strands, and island name (if present) were also identified. The site position in regard to the identified island, gene name and methylation level differences (delta beta) were also included.

| **chromosome** | **position** | **strand** | **Site Name** | **Islands Name** | **Relation to Island** | **UCSC_RefGene_Name** | **delta_beta** |
| --- | --- | --- | --- | --- | --- | --- | --- |
| chr7 | 150929295 | + | cg15251779 | chr7:150929388-150930151 | N_Shore | CHPF2 | 0.065 |
| chr7 | 116404877 | - | cg01297639 |  | OpenSea | MET;MET | -0.042 |
| chr1 | 2536647 | + | cg09105687 | chr1:2537671-2537886 | N_Shore | MMEL1 | -0.034 |
| chr7 | 81320956 | + | cg05548492 |  | OpenSea | LOC100128317 | -0.037 |
| chr3 | 105405658 | + | cg08526006 |  | OpenSea | CBLB | 0.034 |
| chr11 | 121986736 | - | cg27262870 |  | OpenSea | LOC399959;BLID;BLID | -0.034 |
| chr9 | 109170630 | + | cg10544367 |  | OpenSea |  | -0.031 |
| chr6 | 37292135 | + | cg08455089 |  | OpenSea | TBC1D22B | -0.036 |
| chr3 | 58060875 | - | cg24181728 |  | OpenSea | FLNB | -0.032 |
| chr4 | 30920608 | - | cg07022241 |  | OpenSea | PCDH7;PCDH7 | -0.040 |
| chr10 | 270768 | - | cg11324910 |  | OpenSea | ZMYND11 | 0.038 |
| chr11 | 107578208 | - | cg15603354 |  | OpenSea | SLN | 0.035 |
| chr11 | 66532827 | + | cg04049542 | chr11:66529656-66529896 | S_Shelf | C11orf80 | 0.038 |
| chr8 | 94892613 | - | cg10558233 |  | OpenSea |  | 0.032 |
| chr17 | 41137682 | - | cg01910579 |  | OpenSea | RUNDC1 | 0.059 |
| chr10 | 132942686 | + | cg06938601 |  | OpenSea | TCERG1L | -0.102 |
| chr17 | 73399189 | + | cg25589929 | chr17:73401217-73401988 | N_Shelf | GRB2 | 0.030 |
| chr4 | 177575257 | - | cg08320989 |  | OpenSea |  | -0.032 |
| chr2 | 135685009 | - | cg19168673 |  | OpenSea | CCNT2 | 0.033 |
| chr10 | 46991996 | - | cg08821715 | chr10:46992447-46993295 | N_Shore |  | 0.036 |
| chr12 | 99289904 | + | cg10378741 | chr12:99288222-99289374 | S_Shore | ANKS1B | 0.032 |
| chr6 | 139093858 | - | cg10286454 | chr6:139094449-139095009 | N_Shore | CCDC28A;LOC100507462 | -0.031 |
